# P02-021 - Atypical CAPS consequence of novel NLPR3 mutations

**DOI:** 10.1186/1546-0096-11-S1-A128

**Published:** 2013-11-08

**Authors:** E Gonzalez-Roca, G Espinosa, J Bartra, E Ruiz-Ortiz, J Rius, S Plaza, J Yague, JI Arostegui

**Affiliations:** 1 Immunology Service, Hospital Clinic, Barcelona, Spain; 2 Autoimmune Department, Hospital Clinic, Barcelona, Spain; 3 Allergy Department, Hospital Clinic, Barcelona, Spain

## Introduction

Cryopyrin-associated periodic syndromes (CAPS) are a group of dominantly inherited disorders caused by gain-of-function *NLRP3* mutations. These disorders represent different degrees of severity of a same disease being familial cold autoinflammatory syndrome the milder form, Muckle-Wells syndrome an intermediate form and chronic infantile neurologic cutaneous and articular syndrome the severest form. Overlapping phenotypes among these diseases have been also reported. Here we describe two different Spanish families with atypical presentation for CAPS. The initial *NLRP3* screening, which only included the analysis of exon 3, was negative. However, the complete gene analysis revealed two novel missense mutations in exon 1 and 8, respectively.

## Case report

The patient 1 is a 36 year-old woman who suffered from recurrent episodes of uveitis since 2011 and from progressive neurosensorial hypoacusia. She did not refer other symptoms related to CAPS. There was familial history of hypoacusia in her sister, mother and maternal grandmother, suggesting a dominant inheritance pattern for this trait. The *NLRP3* analyses revealed a heterozygous A-to-G transition in the exon 1 of *NLRP3* (at c.146 position), which provokes the missense histidine-to-arginine variant at residue 49 (p.H49R) of cryopyrin.

The propositus of second family is an adult man who referred a pharmacological allergy, and a skin rash in hands, arms and auricular pavilions that apparently appeared in winter and after cold exposure. These data suggested to perform a *NLRP3* analysis that revealed a heterozygous point C-to-T transition at c.2885 nucleotide position, on the exon 8, that provokes the threonine to metionine amino acid change in residue 952 (p.T952M) of cryopyrin.

## Discussion

Most of disease-causing *NLRP3* mutations are located on the exon 3. Here we describe two novel *NLRP3* mutations located on exons 1 and 8, that were identified in patients with atypical presentations for CAPS. In the first family, the detected missense p.H49R mutation represents the first detected mutation identified in the exon 1 of the gene, affecting the PYD domain of cryopyrin. Interestingly, the patient with this mutation was affected by recurrent episodes of uveitis and neurosensorial hypoacusia that appeared during the adulthood, with no other clinical features of CAPS. In the second family, the p.T952M mutation was detected on the exon 8 of the *NLRP3* gene and represents the first and unique mutation identified to date in this exon. These cases highlight the relevance to perform a complete *NLRP3* analyses in potential candidates, including those patients that could refer atypical presentations for CAPS in terms of age at disease-onset or clinical features.

## Disclosure of interest

None declared.

